# piRNA-823 Is a Unique Potential Diagnostic Non-Invasive Biomarker in Colorectal Cancer Patients

**DOI:** 10.3390/genes12040598

**Published:** 2021-04-19

**Authors:** Norhan A. Sabbah, Wael M. Abdalla, Walid A. Mawla, Nagla AbdAlMonem, Amal F. Gharib, Ahmed Abdul-Saboor, Abdallah S. Abdelazem, Nermin Raafat

**Affiliations:** 1 Medical Biochemistry Department, Faculty of Human Medicine, Zagazig University, Zagazig 44523, Egypt; nerminraafat@gmail.com; 2 General Surgery Department, Faculty of Human Medicine, Zagazig University, Zagazig 44523, Egypt; dr.wael_awad@yahoo.com (W.M.A.); Walidabdelmawla@gmail.com (W.A.M.); 3 Tropical Medicine Department, Faculty of Human Medicine, Zagazig University, Zagazig 44523, Egypt; DrNaglahyh@yahoo.com; 4 Department of Clinical Laboratory Sciences, College of Applied Medical Sciences, Taif University, Taif 26513, Saudi Arabia; dr.amal.f.gharib@gmail.com; 5 Clinical Pathology Department, Faculty of Human Medicine, Zagazig University, Zagazig 44523, Egypt; aamoussa@medicine.zu.edu.eg; 6 Medical Biochemistry Department, Faculty of Human Medicine, Suez University, Suez 43514, Egypt; dr_abdo.eldeeb@yahoo.com

**Keywords:** colorectal, cancer, piR-823, biomarkers, CRC, piRNAs

## Abstract

Early detection of colorectal cancer (CRC) is the most important factor in deciding its prognosis, so the need to develop an accurate screening test is a must. P-element induced wimpy testis (PIWI) RNA-823 (piR-823) is one of the first piRNAs recognized to be linked to malignancy. We aimed to investigate the expression levels of piR-823 in both serum and tissues of colorectal cancer patients and the ability to use its serum level as a non-invasive diagnostic biomarker to detect colorectal cancer. We determined piR-823 expression levels in 84 serum samples of CRC patients, 75 serum samples of healthy controls, and biological specimens obtained from the 84 patients with colorectal cancer from both the tumor tissues and the normal neighboring tissues using quantitative real-time reverse transcriptase-PCR. We showed that piR-823 had significantly higher serum and tissue expression levels in CRC patients compared to the controls. We observed a significant positive correlation between piR-823 serum levels and the staging of CRC, with significantly higher levels exhibiting advanced stages of CRC (III and IV). This translates into poorer differentiation and lymph node metastasis. The receiver operating characteristic curve (ROC curve) test showed 83.3% sensitivity and 89.3% specificity at a cut-off value of >5.98-fold change, with an area under the curve of 0.933 (*p* < 0.0001) concerning the ability of piR-823 in diagnosing patients with colorectal carcinoma. piR-823 expression is upregulated in colorectal cancer patients’ serum and tissues, and it can be used as a diagnostic noninvasive biomarker for CRC.

## 1. Introduction

One of the most common causes of death in patients with cancer is colorectal cancer (CRC) [1]. It is the third most diagnosed cancer in men and the second in women [1]. The prevalence of CRC is different all over the world, with a higher incidence in more developed countries (40%) [1]. New Zealand and Australia had reported the highest incidence rates (44.8% in the male and 32.2% in the female) but Western Africa was reported the lowest one (4.5% in the male and 3.8% in the female) [1]. The incidence rate of CRC in Egypt is 5.8% in males and 6.2% in females [2]. Egypt is considered the highest country worldwide regarding the early beginning of CRC, as 35% of CRC patients in Egypt were under forty years. CRC Egyptian patients below the age of 30 years were reported to have three times increased risk to die within five years when compared to CRC patients with the age over fifty years [3]. Patients’ prognosis mostly depends on the TNM staging at the start of the diagnosis besides the feasibility of any possible surgical solution, which is likely to occur only if the disease is localized [4].

To reduce the mortality rate and make the treatment more successful, we must detect CRC as early as possible [1]. Despite all the progress achieved in the screening of CRC, its prognosis is still bad. The standard screening method to detect CRC early is colonoscopy [5]. Colonoscopy is an invasive procedure, so some people avoid doing it because they fear its complications [6]. The other CRC screening investigations such as computed tomography colonography (CT colonography) and fecal occult blood test (FOBT) either have low specificity, sensitivity, or an elevated cost [7]. Hence, the development of an accurate, simple noninvasive blood-based test is of great need that could improve the screening rates.

New molecular markers such as DNA [8], proteins [9], mRNA [10], or microRNAs [11,12] have shown a great potential to be an accurate screening test.

Small non-coding RNAs (ncRNAs) function as key regulators of gene expression and can be simply measured in blood samples [13,14]. One of the small ncRNAs molecules is P-element induced wimpy testis (PIWI)-interacting RNAs (piRNAs), with 24–32 nucleotides long, and are associated with PIWI proteins, which are from Argonaut proteins subfamily [15]. Within the eukaryotic genome over 20,000 piRNAs have been identified [16,17]. piRNAs are expressed in the germline [18] and somatic tissues [19]. PIWI/piRNA complex formed by binding of piRNAs to PIWI proteins, where piRNAs apply for a silencing role in the PIWI-dependent transposon, genome rearrangement, gene and protein regulation, germ stem-cell maintenance, and epigenetic regulation [20]. piRNAs and PIWI proteins were proved to have an important role in the control, prognosis, and treatment of many types of tumors. Making them eligible to be used as therapeutic and diagnostic tools for various types of cancer [21].

Many studies showed that a variety of cancers have an aberrant expression of piRNA or PIWI protein including gastric cancer [22], multiple myeloma [23], liver cancer [24], lung cancer [25], breast cancer [26], and bladder cancer [27] correlating it with tumorigenesis.

PIWI RNA-823 (piR-823) was one of the first piRNAs recognized to be linked to malignancy [22]. piR-823 was identified in white blood cells [28], cancer cell lines, and blood plasma [29,30]. It was reported to be involved in regulating tumor cell growth, and its level fluctuates in many cancers, including gastric cancer tissue and myeloma cells [22,23].

Because of the above evidence, we investigated the expression levels of piR-823 in both serum and tissues of CRC patients to assess its ability to use its serum level as a non-invasive diagnostic marker to detect colorectal cancer.

## 2. Materials and Methods

### 2.1. Study Design

This study was approved by the International Review Board (IRB) and the ethical committee, Faculty of Medicine, Zagazig University, the reference number is (Zu-IRB#3245/10-5-2019) and we received a signed written consent from all patients included in the study. Eighty-four CRC patients were recruited from the Department of General Surgery, between June 2019 and September 2020.

Clinicopathological characteristics are mentioned in Table 1. Patients included were undergoing colonoscopy and were proved to have adequate hepatic, renal, cardiac, and respiratory functions. Colorectal adenomas (CRAs) and CRCs were confirmed histopathologically. Tumor typing and grading were based on the published criteria of the WHO 2010 [31]. We based the staging of CRC cases on the TNM staging systems [32]. Patients with inflammatory bowel diseases, history of other tumors in the previous or consistent periods, preoperative chemotherapy or radiotherapy, or refusal of the patients or their guardians to take part were excluded from the study.

Seventy-five healthy subjects were included in our study, who were both sex and age matched with CRC cases and were considered the control group in case of the blood samples. They were selected randomly from different departments with no family history and no cancer discovered recently or in the past and after undergoing colonoscopy for any other causes or after taking their consents.

### 2.2. Sampling

Five ml of whole blood were collected in sterilized tubes from patients and controls, then were left to complete clot and centrifuged at 3000× *g* to separate the serum, then the samples were stored at −20 °C.

We carried this case-control study in the Department of Medical Biochemistry and the ZSMRC (Zagazig scientific medical research center, Zagazig, Egypt).

### 2.3. Serum Carcinoembryonic Antigen (CEA) Level and Carbohydrate Antigen 19-9 (CA19-9) Level

Serum CA19-9 was measured by using commercial Eliza kit (Abbott Laboratories, Chicago, IL, USA) Catalog No. ab108642 while measurement of serum CEA levels was performed by electro-chemiluminescence immunoassay (Roche, Mannheim, Germany) Catalog NO. E1-207 using a double-antibody sandwich enzyme-linked immunosorbent assay, cut-off values used were 5 μg/L for CEA and 35 U/mL for CA19-9. A result was deemed positive when the marker serum level was higher than the cut-off value.

### 2.4. Total RNA Extraction

Serum total RNA was extracted using the miRNeasy Serum\Plasma kit (Catalog no. 217184; Qiagen, Hilden, Germany).

In tissue samples, the previously frozen specimen was homogenized by the homogenizer. Total RNA was extracted using miRNeasy Mini kit (Catalog no. 217004; Qiagen, Germany). For evaluating the RNA quality, the A260/A280 ratio was measured and analyzed using the Nano Drop^®^ ND–1000 Spectrophotometer (Nano Drop Technologies, Wilmington, DE, USA).

### 2.5. Quantitative Real-Time Polymerase Chain Reaction Expression of piR-823

The total RNA was reverse transcribed using miScript RT Kit (Qiagen, Germany) where a mixture of 1 μL miScript Reverse Transcriptase Mix, 4 μL miScript RT buffer, and 2 μg total RNA was formed, then incubated at 37 °C for 60 min.

We used the miScript SYBR Green PCR Kit (Qiagen, Germany) for performing the polymerase chain reaction (PCR) on Stratagene, MX3000P quantitative PCR System (Agilent Technologies) and analyzed using the MxPro QPCR Software (Agilent Technologies). The PCR mixture (20 μL) was formed of 2×QuantiTect SYBR Green PCR Master Mix (10 μL), RT product (4 μL), 10×miScript Universal Primer (1.5 μL) (downstream PCR primer for small RNA; Qiagen), 10× piR-823 (GenBank: DQ571031.1) upstream PCR primer (1 μL), piR-823 upstream primer sequence 5′-AGCGTTGGTGGTATAGTGGT-3′, and distilled water (3.5 μL) [33]. The conditions of the PCR reaction were: 15 min at 95 °C, then 40 cycles of (94 °C for 15 s, 60 °C for 30 s, and 70 °C for 30 s). piR-823 expression level was normalized by using U6 as an internal control. The amount of piR-823 expression change, in patients relative to controls, was evaluated by the ΔΔCt method [34].

## 3. Statistical Analysis

Data analysis was conducted with SPSS version 15.0 (Statistical Package for the Social Science, Chicago, IL, USA). Quantitative data were presented as mean ± SD (parametric variable), median (non-parametric), frequency and percent (categorical variable), chi-squared test, Student’s *t*-test, and one-way analysis of variance were applied when needed. A difference was significant if the *p*-value is <0.05. *p*-value was adjusted after Bonferroni correction. The capability of piR-823 expression to differentiate between case and control was calculated by plotting the receiver operating characteristics curve (ROC curve), which relates the true positive (sensitivity) to the false positive (specificity) and by the computing area under the curve (AUC).

## 4. Results

All the clinicopathological characteristics of the patients and the controls were listed in Table 1, where there were no significant differences concerning age and gender, with CEA levels the number of positive cases was 59 (70.24% of the cases) while the number of positive cases of CA 19-9 was 40 (47.62%).

We determined piR-823 expression levels in the serum samples of 47 male and 37 female CRC patients of age 59.12 ± 2.61, 40 male and 35 female healthy controls of age 61.22 ± 3.31, and in biological tissues specimens obtained from the 84 patients with colorectal cancer including core tumor tissues and neighboring normal healthy tissues to detect the expression level of piR-823 and test the feasibility of using it as a tumor marker.

We found that piR-823 is significantly upregulated in the serum of patients with CRC 6.2 ± 2.3-fold-change when compared to healthy controls (1.0 ± 0.2) (*p* < 0.001). We observed also a highly significant difference in piR-823 expression levels between tumors 3.4 ± 1.9-fold-change and their corresponding adjacent normal tissue samples (1.0 ± 0.4) (*p* < 0.001) (Table 2).

When we investigate the association of piR-823 expression with the clinicopathological features of the CRC cases, we found that its expression in tissues showed no association with gender, tumor size, tumor location, and lymph node metastasis (*p* > 0.05); however, it associated with the staging of tumors and the differentiation degree, with a significantly elevated expression in poorly differentiated tumor tissues (3.9 ± 1.4) (*p* < 0.05) and stages III and IV (4.4 ± 1.5) (*p* < 0.001) (Table 3).

We detected highly significant levels of serum piR-823 expression associated with advanced clinical stages of CRC (III and IV) (7.5± 1.5) (*p* < 0.001), poor differentiation (9.2 ± 1.9) (*p* < 0.001), and lymph node metastasis (6.8 ± 2.4) (*p* < 0.05) (Table 3).

**Figure 1:**
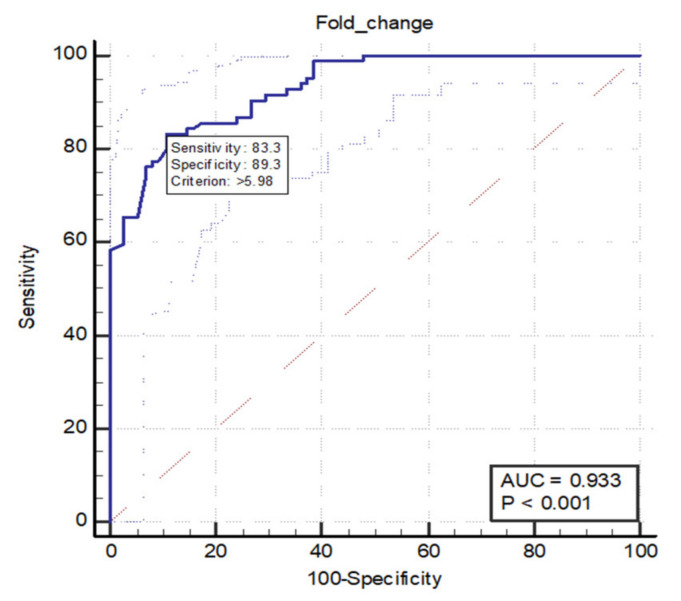
Receiver operating characteristics curve (ROC curve) of performance of piR-823 in diagnosing patients with colorectal carcinoma. The test shows 83.3% sensitivity and 89.3% specificity at a cut-off value of >5.98-fold change, with an area under the curve of 0.933 (*p* < 0.0001).

When we plotted the ROC curve to investigate the ability of piR-823 in diagnosing patients with colorectal carcinoma. The test showed 83.3% sensitivity and 89.3% specificity at a cut-off value of >5.98-fold change, with an area under the curve of 0.933 (Figure 1).

**Figure 2:**
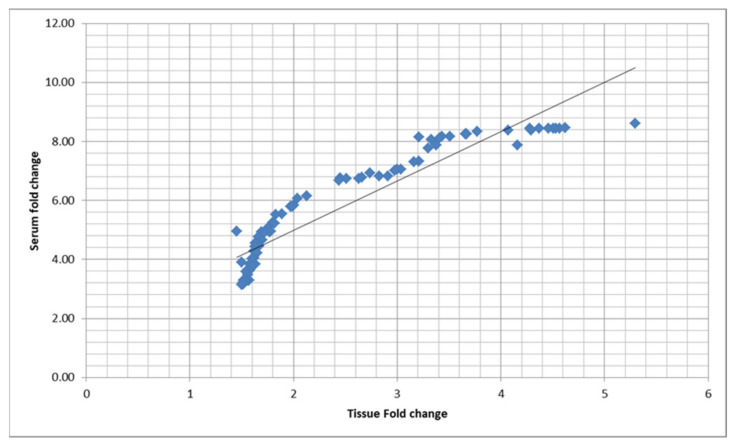
Correlation between serum and tissue expression of piR-823 measured in fold change. The curve shows a strong positive correlation with a correlation coefficient of 0.9285, *p* < 0.0001.

Correlation study of the relationship between expression of piR-823 in serum of colorectal carcinoma patients and the affected tissue shows a strong positive correlation, with a correlation coefficient of 0.9285, *p* < 0.0001 (Figure 2), proving that increased piR-823 in tissues correlated with its increase in serum so there is no need to the invasive colonoscopy which can be replaced by noninvasive blood sampling.

These results proved that piR-823 is upregulated in colorectal cancer, and its serum level can act as a diagnostic biomarker in CRC patients.

## 5. Discussion

Colorectal cancer ranks third in morbidity and second in mortality among different cancers worldwide [35]. The development in using colonoscopy has improved the outcome of detection and resection of colorectal polyps. Still, no significant improvement has been observed in the prognosis of colorectal cancer since 1997 [36]. For an improvement to occur, we should rethink conventional detection and treatment methods and instead use more selective therapies guided by new discoveries in tumor biomarkers.

One of those new discoveries is the significant link between piRNA and PIWI expression levels and tumors ongoing research is still assessing its usefulness in targeted therapy [23,24,25,27].

piRNAs promising outlook as an early tumor biomarker can be attributed to its relative stability compared to miRNAs because of the presence of PIWI protein, which is thought to have a protective function [37]. piR-823 was among the first piRNAs reported having this link, directing many studies to test its usefulness as a biomarker, including this one [22,23,28,29].

We showed that piR-823 had a higher serum level in CRC patients compared to healthy controls moreover we observed a correlation between piR-823 serum levels and the staging of CRC, with higher levels exhibiting advanced stages of CRC (III and IV), this translates into a poorer differentiation and lymph node metastasis. As far as we know, we were the first to measure piR-823 in CRC patients’ serum and correlate it with its diagnosis, however our results are in line with other studies which focused mainly on piR-823 tissue level in CRC patients, notably Yin et al. in 2017 [38] who was the first study to point out the piR-823 upregulation in CRC tissues compared to its normal counterparts. We found that piR-823 expression in tissues, like its serum level, was correlated with differentiation and staging of colorectal cancer. A correlation study of the relationship between expression of piR-823 in serum of colorectal carcinoma patients and the affected tissue shows a strong positive correlation. Proving that increased piR-823 in tissues correlated with its increase in serum, emphasizing that monitoring the expression of piR-823 in blood could be a non-invasive and easy diagnostic procedure.

Few mentions of altered piRNAs levels in CRC were found in the scientific literature, with some promoting tumor growth and others having a suppressor role. CRC cell growth had significantly sped up with high levels of piR-1245 [39]. The expression of piR-1245 is higher in colorectal tumor tissues with lymph node metastasis, poor differentiation, distant metastasis, advanced T stage, and low overall survival (OS), and piR-1245 increase the growth of CRC cell by promoting invasion, migration, and preventing apoptosis however, it was not measured in serum [39]. While piR-54265 was found to aid CRC cell proliferation and metastasis and meanwhile upregulated in CRC tissues [40]. It was higher in shorter progression-free survival and OS, its increase caused chemoresistance by increasing 5-FU and oxaliplatin half maximal inhibitory concentrations. The same as piR-1245, it promoted CRC growth by inhibiting apoptosis mainly by PIWIL2/STAT3/p- SRC complex creation, but its serum level was not measured [40].

piR-015551 has abnormal expression in CRC tissues according to its variants, while its rs11776042 variant (thymine to cytosine; T > C) alters piRNA’s secondary structure, which changes piRNA influences on colorectal cancer development [41], meanwhile piR-5937 is downregulated in CRC and is used to identify cancer patients from healthy ones [42].

piR-823 has been observed to have roles in cancers other than CRC albeit with different mechanisms, one study concluded that piR-823 may have a tumor suppressive role in gastric cancer as it was markedly downregulated [22], similarly it was found to be downregulated in renal cell carcinoma (RCC), but paradoxically it was positively correlated with worse prognosis, suggesting a more complex mechanism in RCC than previously thought [43]. On the contrary, extracellular vesicles associated with multiple myeloma exhibited higher levels of piR-823 than normal, and was positively correlated with the clinical stages of myeloma [37].

piR-823 role in CRC development is complex and includes different mechanisms, it alters heat shock factor 1 (HSF1) by a post translational mechanism leading to a suppression in cell apoptosis as well as an enhancement in proliferation [38]. piR-823 promotes HSF1 phosphorylation at Ser326 leading to increased expression of heat shock proteins which may explain its role as a strong driver for oncogenesis [38]. Another mechanism is the phosphorylation of STAT3 by PIWI/piRNA complex2 and the activation of STAT3/BCL-xl/cyclinD1 signaling pathway, which may induce cyclin-dependent kinase inhibitor (CDKI) expression and control G1 phase progression [44]. This has therapeutic implications as piR-823 inhibitor leads to G1 phase stagnation and downregulate cyclin D1 and CDK4 [45], consequently reversing piR-823 role in CRC cell proliferation, and thus it inhibits CRC proliferation and induces cell apoptosis, further cementing piR-823 role in targeted therapy [38]. The downstream targets of piRNA-823 include DNA methyltransferases (DNMTs) genes as DNMT1, DNMT3A, and DNMT3B which maintain methylation of DNA leading to regulation of gene expression, DNMTs gene expression is increased by piR-823, which encouraged adenomatous polyposis coli (APC) gene methylation thus causing Wnt signaling activation and provoking cancer cell stemness, on the other hand piRNA-823 was proved to promote carcinogenesis by regulating cancer stem cells [46].

Sellitto et al. studied the PIWIL/piRNA pathway in COLO 205 colorectal cancer cells, which express significant levels of this protein, they demonstrated PIWIL1 is in a Nuage-like assembly found in the cell perinuclear part where a noteworthy portion of methylated piRNAs was expressed in an active form. They revealed also that many piRNAs can be found loaded into PIWIL1 forming complexes comprising their target mRNAs. The matured transcripts linked to the PIWIL/piRNA complex coded molecular mechanisms’ key regulatory proteins which took part in CRC tumorigenesis, implying that the PIWI/piRNA complex can take part in establishing or maintaining the CRC clinico-pathological features [47].

As piRNAs mechanisms include mainly the upstream of multiple signaling pathways and regulatory networks, they represent a new opportunity in early cancer screening and subsequent treatment [37].

piR-823 show promise as novel complementary tumor markers for cancer if further confirmed by different expression of piRNA in serum of cancer patients compared to controls, and its significant association with clinical tumor stages and aggressive biological behavior. It also represents a convenient screening tool, as blood sampling is a non-invasive diagnostic method and is already used in clinical settings. Several studies deduced the feasibility of using piR-823 as a preferential biomarker for screening cancer using blood samples as in gastric cancer [22], RCC [43], and multiple myeloma [37]. They all agreed that piR-823 is a brilliant cancer biomarker which not only used for diagnosis but also for screening and prognosis.

## 6. Conclusions

piR-823 expression is upregulated in colorectal cancer patients’ serum and tissues and it can be used as a diagnostic biomarker for CRC however further studies are needed to confirm this and reveal its mechanism of action to be used in future therapy.

## Figures and Tables

**Table 1:** Clinicopathological features of the studied groups.

Parameter	Cases	Control
	No. 84(%)	No. 75(%)
Age ± (Mean SD)	59.12 ± 2.61	61.22 ± 3.31
Gender number (%)		
- Male	47 (56)	40 (53.3)
- Female	37 (44)	35 (46.7)
Tumor location number (%)		
- Distal	52 (62)	
- Proximal	32 (38)	
TNM staging number (%)		
- Stage I	19 (22.62)	
- Stage II	25(29.76)	
- Stage III	21 (25)	
- Stage IV	19 (22.62)	
Tumor grading number (%)		
- Grade 1	24 (28.57)	
- Grade 2	43 (51.19)	
- Grade 3	17 (20.24)	
Tumor differentiation number (%)		
- Well	22 (26.19)	
- Moderate	38 (45.24)	
- Poor	24 (28.57)	
Lymph node metastasis number (%)		
- Negative	21 (25)	
- Positive	63 (75)	
CEA number (%)		
- Negative	25 (29.76)	
- Positive	59 (70.24)	
CA 19-9, number (%)		
- Negative	44 (52.38)	
- Positive	40 (47.62)	

**Table 2:** P-element induced wimpy testis (PIWI) RNA-823 (piR-823) expression in colorectal cancer cases (CRC) and control subjects.

Parameter	piR-823 Level	*p* Value
Serum of healthy control	1.0 ± 0.2	<0.001 *
Serum of CRC cases	6.2 ± 2.3	
Adjacent normal tissues of CRC cases	1.0 ± 0.4	<0.001 #
Tumor tissues of CRC cases	3.4 ± 1.9	

piR-823 level is in fold change relative to control, statistically highly significant difference (*p* ≤ 0.001), *p*-value for *t* test, * when serum of CRC cases compared to control, and # when tumor tissue compared to normal tissues.

**Table 3:** Association between piR-823 expression and clinicopathological characteristics in colorectal cancer cases.

		Tumor Tissue of CRC Cases		Serum of CRC Cases	
Parameter	No. of Cases (%)	piR-823 (Mean ± SD)	*p* Value	piR-823 (Mean ± SD)	*p* Value
Gender			0.35		0.438
- Male	47 (56)	3.3 ± 1.8		6.0 ± 2.2	
- Female	37 (44)	3.7 ± 2.1		6.4 ± 2.5	
Tumor Location			0.51		0.334
- Distal	52 (62)	3.3 ± 1.9		5.8 ± 2.4	
- Proximal	32 (38)	3.6 ± 2.2		6.3 ± 2.1	
TNM staging.			** <0.001		** <0.001
-Stage I, II	44 (52.38)	2.2 ± 2.9		5.3 ± 3.1	
-Stage III, IV	40 (47.62)	4.4 ± 1.5		7.5 ± 1.5	
Tumor Grading			0.771		0.653
- Grade 1, 2	67 (79.76)	3.4 ± 2.7		6.1 ± 2.5	
- Grade 3	17 (20.24)	3.6 ± 1.6		6.4± 2.2	
Differentiation			* 0.042		** <0.001
-Well, Moderate	60 (71.43)	2.9 ± 2.2		3.5 ± 2.7	
-Poor	24 (28.57)	3.9 ± 1.4		9.2 ± 1.9	
Lymph node metastasis			0.454		* 0.031
- Negative					
- Positive	21 (25)	3.2 ± 1.8		5.5 ±2.2	
	63 (75)	3.6 ± 2.2		6.8 ± 2.4	
CEA			0.525		0.7
- Negative	25 (29.76)	3.6 ± 1.9		6.1 ±2.7	
- Positive	59 (70.24)	3.3 ± 2.0		6.3 ± 1.9	
CA 19-9			0.39		0.118
- Negative	44 (52.38)	3.7 ± 1.2		5.8 ± 2.4	
- Positive	40 (47.62)	3.3 ± 2.8		6.6 ± 2.2	

piR-823 level is in fold change, *p*-value for *t*-test, * statistically significant difference (*p* ≤ 0.05), ** statistically highly significant difference (*p* ≤ 0.001).

## Data Availability

Data available on request due to privacy/ethical restrictions.
